# A Reliable and Effective UPLC-MS/MS Method for the Determination of Oprozomib in Rat Plasma

**DOI:** 10.1155/2023/3678599

**Published:** 2023-07-11

**Authors:** Xia Li, Ya-nan Liu, En Zhang, Ren-ai Xu, Tingyong Yang, Shunbin Luo

**Affiliations:** ^1^Clinical Laboratory, The Affiliated Lihuili Hospital, Ningbo University, Ningbo, Zhejiang, China; ^2^The First Affiliated Hospital of Wenzhou Medical University, Wenzhou, Zhejiang, China; ^3^Chongqing Key Laboratory of Translational Research for Cancer Metastasis and Individualized Treatment, Chongqing University Cancer Hospital, Chongqing, China; ^4^The People's Hospital of Lishui, Lishui, Zhejiang, China

## Abstract

Oprozomib, as a second-generation orally bioavailable protease inhibitor (PI), is undergoing clinical evaluation for the treatment of haematological malignancies. In relapsed refractory multiple myeloma (RRMM) patients, oprozomib has shown good efficacy as a single agent or combination therapy. In this experiment, our purpose was to validate a sensitive and rapid method for the determination of oprozomib concentration in rat plasma by ultraperformance liquid chromatography tandem mass spectrometry (UPLC-MS/MS). The samples were treated with acetonitrile as the precipitant and separated by gradient elution using a Waters Acquity UPLC BEH C18 column (2.1 mm × 50 mm, 1.7 *μ*m). Using the selective reaction monitoring (SRM) method, the measurement was finished with the ion transitions of *m/z* 533.18 ⟶ 199.01 for oprozomib and *m/z* 493.03 ⟶ 112.03 for tepotinib (internal standard, IS), respectively. Meanwhile, acetonitrile and 0.1% formic acid aqueous solution were used as the mobile phase, and the flow rate was 0.3 mL/min. The lower limit of quantification (LLOQ) of the method was 1.0 ng/mL, and the linear relationship was good in the range of 1.0–100 ng/mL. In addition, the precision of four concentration levels was determined with the values of 3.1–7.3% and the accuracy was from −14.9% to 12.9%. Moreover, the recovery was determined to be from 85.1% to 96.1%, and the values of matrix effect were no more than 110.4%. The optimized UPLC-MS/MS method was also suitable for the pharmacokinetic study of rats after a single oral administration of 21 mg/kg oprozomib.

## 1. Introduction

Following lymphoma, multiple myeloma (MM) is the most common hematologic malignancy, which is a neoplasm of clonal plasma cells and belongs to postgerminal lymphoid B-cell lineage [1, 2]. At the present medical level, MM is still not completely cured, but with the development of modern medicine, protease inhibitor (PI), immunomodulatory drugs, monoclonal antibody (MoAb), stem cell transplantation therapy, and other drugs and treatment methods appear for MM patients with the treatment and prolong the survival of a new hope [3].

**Figure 1:**
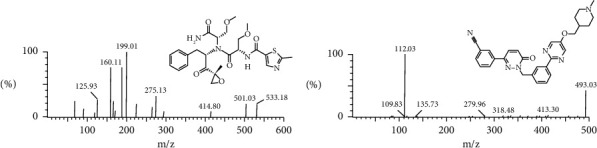
Mass spectra of oprozomib (a) and tepotinib (IS), (b) in this study.

PIs have been proved to be an effective and common treatment for MM [4]. Oprozomib (Figure 1(a)), as a second-generation orally bioavailable PI, is undergoing clinical evaluation for the treatment of haematological malignancies [5, 6]. In relapsed refractory multiple myeloma (RRMM) patients, oprozomib has shown good efficacy as a single agent or combination therapy. The pharmacokinetic and pharmacodynamic characteristics of oprozomib have previously been studied in phase I studies in solid tumor patients, which demonstrated oral bioavailability and showed dose-dependent proteasome inhibition [5, 7, 8].

In most cases, oprozomib is used in combination with other agents, including first-generation agents, when treating MM/RRMM, and drug-drug interactions (DDIs) have to be considered [9]. Studies have shown that physiology-based pharmacokinetic (PBPK) models have been well used to promote the design and extrapolate DDIs [10]. Although good progress has been made in simulating the interaction between oprozomib and other drugs based on PBPK model, *in vitro* metabolism study of oprozomib has shown that it was unlikely to be affected by coadministered cytochrome P450 (CYP) [11].

In order to explore the pharmacokinetics and DDIs of oprozomib *in vivo*, it is necessary to build a sensitive quantitative detection method for oprozomib in biological fluids to provide a basis for subsequent clinical research. However, until now, the method of bioanalysis and determination of oprozomib in biological liquid has not been established. Thus, in this experiment, our purpose was to validate a sensitive and rapid method for the determination of oprozomib concentration in rat plasma by ultraperformance liquid chromatography tandem mass spectrometry (UPLC-MS/MS). The newly established UPLC-MS/MS technique was also suitable for the pharmacokinetics of oprozomib in rats.

## 2. Experimental

### 2.1. Chemical Materials and Reagents

The purity of both oprozomib and tepotinib (used as internal standard, IS, Figure 1(b)) was >98.0% and was provided by Shanghai Chuangsai Technology Co., Ltd. (Shanghai, China). In addition, sodium carboxymethyl cellulose (CMC-Na) was also purchased from Shanghai Chuangsai Technology Co., Ltd. (Shanghai, China). Methanol and acetonitrile supplied by Merck (Darmstadt, Germany) in this study were of liquid chromatography (LC) grade. Moreover, formic acid was of LC grade, which was supplied by Anaqua Chemicals Supply (ACS, American). The ultrapure water used in the experiment was prepared by Milli-Q Water Purification System (Millipore, Bedford, USA).

### 2.2. Animal Experiments

Six Sprague Dawley (SD) rats (weight 200 ± 20 g) were provided by the Laboratory Animal Center of The First Affiliated Hospital of Wenzhou Medical University (Wenzhou, China) and were fed under standard condition, which were temperature 25–28°C, humidity 50–60%, and 12 h light/12 h dark. All the rats were fed on a standard rodent diet, which supplied water and food without limit. Under the rules for the Care and Use of Laboratory Animals, all behaviors and operations in this experiment were reviewed and approved by the Institutional Ethics Committee of The First Affiliated Hospital of Wenzhou Medical University (Zhejiang, China).

Oprozomib was prepared into suspension with 0.5% CMC-Na solution and administered orally to rats at a dose of 21 mg/kg. Before the experiment, six rats were fasted for 12 h, while all of them were allowed to access water unrestrainedly. At different time points of 0.3, 0.7, 1.0, 1.5, 2.0, 3.0, 4.0, 6.0, 8.0, 12, 24, and 48 h, about 0.3 mL of blood samples was taken from the caudal vein and placed in a 1.5 mL Eppendorf (EP) tube containing heparin. Then, the blood samples were centrifuged rapidly at 4000 × *g* at 4°C for 10 min to obtain plasma samples. Subsequently, the treated plasma was stored at −80°C before the further analysis. After sample preparation, the plasma concentration of oprozomib in rats was evaluated and detected by UPLC-MS/MS method. Then, pharmacokinetic parameters of oprozomib in noncompartmental analysis were calculated by DAS software (Drug and Statistics, version 3.0, Shanghai University of Traditional Chinese Medicine, China).

### 2.3. Instrumentations and Analytical Conditions

The UPLC-MS/MS system, coupled with an electrospray ionization (ESI) source (Milford, MA, USA), was composed of the Waters Xevo TQ-S triple quadrupole tandem mass spectrometer and the Waters Acquity UPLC I-Class system (Milford, MA, USA).

In this study, solvent A (0.1% formic acid aqueous solution) and solvent B (acetonitrile) were used as the mobile phase, and oprozomib and IS were separated on an Acquity UPLC BEH C18 Van-Guard precolumn (2.1 × 5 mm, 1.7 *μ*m) and a C18 column (2.1 mm × 50 mm, 1.7 *μ*m). Before linear gradient elution, 10% B was balanced between 0-0.5 min, and elution was performed at a flow rate of 0.30 mL/min as follows: 0.5–1.0 min (B, 10–90%), 1.0–1.4 min (B, 90%), and 1.4–1.5 min (B, 90 − 10%). During instrument analysis, the temperature in the automatic sampler was 10°C, and the column temperature was 40°C. The analysis time was 2.0 min, and the injection volume was 2.0 *μ*L.

Mass spectrometry monitoring was performed using the Xevo TQ-S triple quadrupole tandem mass spectrometer, in conjunction with the Acquity UPLC system for analysis, in positive ion mode for the detection of oprozomib and IS. The selective reaction monitoring (SRM) mode of ion transitions was *m/z* 533.18 ⟶ 199.01 and *m/z* 493.03 ⟶ 112.03 for oprozomib and IS, respectively. The collision energy and cone voltage of oprozomib were 25 eV and 20 V, and those of IS were 15 eV and 30 V. Other experimental conditions for mass spectrometry were: desolventizing temperature (600°C), capillary voltage (2.0 kV), collision gas (0.15 mL/min), tapered gas (200 L/h), and dissolved gas (1000 L/h).

### 2.4. Standard Solutions, Calibration Curves, and Quality Control (QC) Samples

For the preparation of quality control (QC) samples and calibration curves, both oprozomib and IS reserve solutions at a concentration of 1.0 mg/mL were used as the primary solution. A series of working solutions with different concentrations were obtained by gradient dilution of methanol in the raw solution of the tested substance. Subsequently, a working solution (5 *μ*L) was added to blank rat plasma (45 *μ*L), which was equivalent to a 10-fold dilution of working solution. A standard curve with a final concentration range of 1.0–100 ng/mL (1.0, 2.0, 5.0, 10, 20, 50, and 100 ng/mL) was obtained. Similar to the preparation of standard curves, the final concentrations of QC samples were 1.0 (lower limit of quantitation, LLOQ), 2.0 (low quality control, LQC), 40 (medium quality control, MQC), and 80 (high quality control, HQC) ng/mL. The IS working solution with a concentration of 100 ng/mL was obtained by diluting stock solution with methanol. At the beginning of the experiment, all raw and working fluids need to be prepared in advance and stored at −80°C.

### 2.5. Sample Preparation

In preparing all samples, we performed protein precipitation method and acetonitrile was employed as the precipitant. First, 50 *μ*L of plasma sample, in a 1.5 mL EP centrifuge tube, was spiked with 10 *μ*L of IS working solution (100 ng/mL) and mixed for 0.5 min in a vortex. Then, a total of 450 *μ*L acetonitrile was added to precipitate the plasma protein, and the mixture was vigorously vortexed for 1.0 min, followed by a 10 min centrifugation at 13,000 × rpm under 4°C. The next step was to transfer 100 *μ*L of the supernatant into a clean automatic injection flask. Finally, the injection volume was set at 2.0 *μ*L, and a chromatographic system for quantitative analysis was used.

### 2.6. Method Validation

This analysis method followed FDA guidelines for the validation of bioanalytical tests, and the method validation procedures including the calibration curve, stability, matrix effect, LLOQ, precision, and accuracy were fully conducted [12].

In this experiment, in order to detect the selectivity of the method, blank plasma samples from six different rats were analyzed by chromatography to evaluate whether there was endogenous interference at the retention times of both the analyte and IS in the method.

We constructed the standard curve according to the principle of least square method and used the weighting factor of the reciprocal of concentration (1/*x*) to measure its linearity. LLOQ is the lowest concentration of the analyte on the standard curve that can be quantified reliably, with acceptable accuracy and precision. At the LLOQ, the accuracy (relative error, RE, %) is ±20%, and the precision (relative standard deviation, RSD, %) is less than 20%.

The precision and accuracy of QC samples were evaluated by five repeated tests at three different concentration levels for three consecutive days. Under three concentration levels (five samples were made in parallel for each concentration), the recovery rate was calculated by comparing the concentration ratio of the analyte before and after protein precipitation. As well, the response rate of extracted plasma matrix and analyte in pure solution was compared to calculate the matrix effect, and the matrix effect was also measured in five repetitions.

LQC (2.0 ng/mL), MQC (40 ng/mL), and HQC (80 ng/mL) were used for stability evaluation. The specific evaluation contents were divided into the following four parts [13, 14]: one was the stability at room temperature for 3 h and −80°C for 3 weeks as another, the third was the stability of automatic sampler at 10°C for 4 h, and the last was three times of complete freezing and thawing from −80°C to room temperature.

## 3. Results and Discussion

### 3.1. Method Design and Optimization

At the beginning of the study, we optimized the chromatographic conditions and improved the detection sensitivity to establish a simple UPLC-MS/MS method for the determination of oprozomib in rat plasma. As presented in Figure 1, mass spectra of oprozomib and IS were shown. In the process of quality transformation, we used *m/z* 199.01 as the quantitative method of product ions and with *m/z* 112.03 as the IS ion quantitative method. In addition, the experiment used 0.1% formic acid aqueous solution and acetonitrile solution as mobile phase and resulted in excellent separation effect and peak shape.

In the experiment, acetonitrile was used as the protein precipitator when we treated plasma. The method was simple and the experimental time was short. In addition, we also comprehensively evaluated and analyzed the feasibility of this method in the detection of oprozomib from the aspects of accuracy, precision, and stability. Comprehensively, this was a simple and effective detection method.

### 3.2. Method Validation

#### 3.2.1. Selectivity

**Figure 2:**
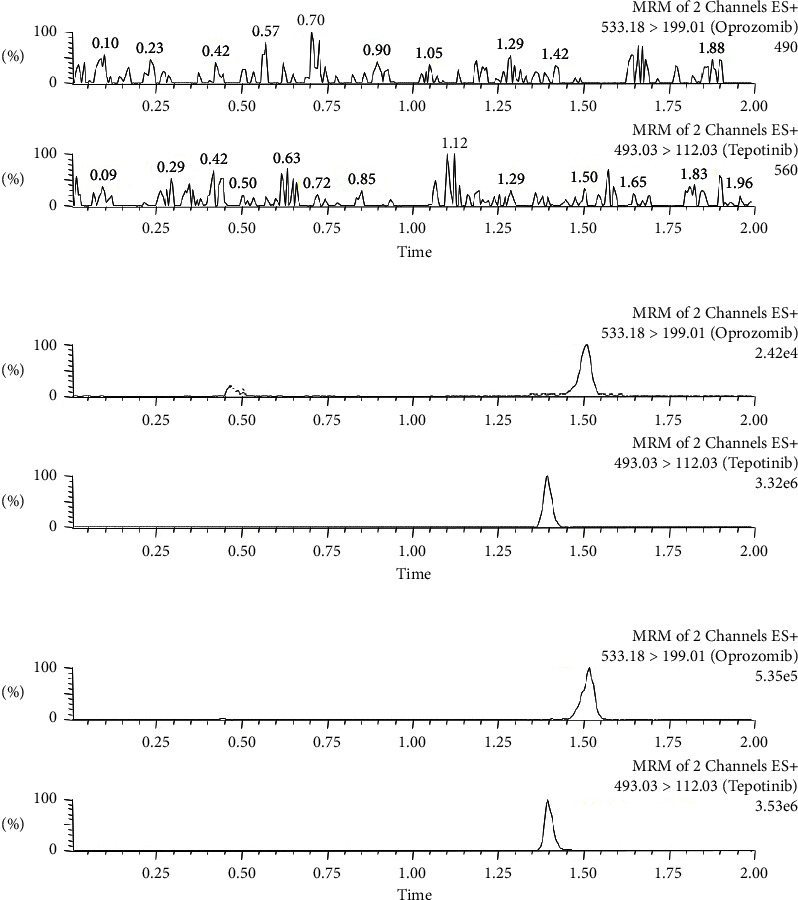
Representative chromatograms of oprozomib and IS in rat plasma: (a) blank plasma; (b) blank plasma spiked with standard solution at LLOQ (1.0 ng/mL) and IS; (c) sample obtained from a rat after oral administration of 21 mg/kg oprozomib.

As shown in Figure 2, the retention times of oprozomib and IS were about 1.52 and 1.39 min, respectively. After comparing the representative SRM chromatograms of blank rat mixed plasma from 6 different sources, blank plasma supplemented with oprozomib and IS at LLOQ concentration level, and experimental plasma samples, no potential interfering substances were found. Based on the results, the method enabled to be used to determine the concentration of oprozomib since it had good selectivity for oprozomib and IS.

#### 3.2.2. Linearity and Sensitivity

The concentration range of the standard curve was 1.0–100 ng/mL, and the linear regression equation between peak ratio (*Y*) and compatible concentration (*X*) was *Y* = 0.00683 × *X* + 0.00259 (*R*^2^ = 0.9993), where good linear relationship met the requirements of methodological detection. The LLOQ of the method was 1.0 ng/mL, where the precision was less than 7.3% and the accuracy was with ±5.3%.

#### 3.2.3. Precision, Accuracy, Recovery, and Matrix Effect

As part of the UPLC-MS/MS method, the precision and accuracy assessments were detected by calculating the QC samples with different concentration levels (*n* = 5) at 1.0, 2.0, 40, and 80 ng/mL for 3 consecutive days.  Table 1 shows that the UPLC-MS/MS method established for quantitative analysis of oprozomib in rat plasma was highly precise and accurate.

In this study, HQC, MQC, and LQC were used to detect the recovery and matrix effect of five repetitions. The means of calculating extraction recovery were used to compare the plasma samples added to QC before protein precipitation with the plasma concentrations added after extraction. Using this method, the extraction recovery of three concentration levels ranged from 85.1% to 96.1% (Table 2). The matrix effect was calculated by IS normalization method, and no matrix effect was observed in the experiment to affect the determination of the analyte in plasma (Table 2), suggesting that this method was reliable and effective.

#### 3.2.4. Stability

The stability of oprozomib in rat plasma under different conditions was investigated by stability experiments. In the experiment, plasma samples added with oprozomib were stored or processed under four conditions: at least 3 weeks at −80°C, after three complete freezing (−80°C)/thawing (room temperature) cycles, at least 4 h in automatic sampler (10°C), and under room temperature at least 3 h. Stability test results showed that the drug was stable in routine experiments as shown in Table 3.

### 3.3. Animal Study

**Figure 3:**
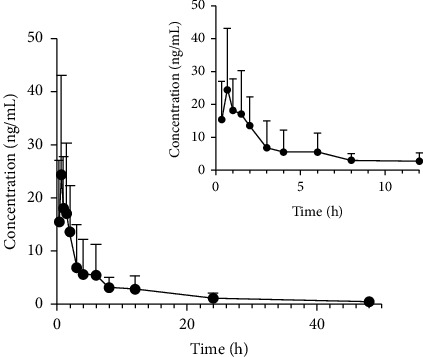
Mean plasma concentration-time curves of oprozomib in rats after oral administration of oprozomib at a single dose of 21 mg/kg (*n* = 6).

The UPLC-MS/MS method established in this study was suitable for the determination of plasma drug concentration in rats after a single oral dose of 21 mg/kg oprozomib. The average plasma concentration time curve of oprozomib in rats is shown in Figure 3. The main pharmacokinetic parameters of oprozomib were analyzed by noncompartmental model and are summarized, as shown in Table 4. The peak time (*T*_max_) of the drug in rat plasma was about 1.1 ± 0.3 h and the half-life (*t*_1/2_) was 13.8 ± 8.3 h. Considering that only 6 rats were used in our animal experiment, if want to determine the pharmacokinetics curve of oprozomib more accurately, we need to expand the number of animal experiments and conduct further research.

## 4. Conclusion

Above all, we developed a simple and reliable UPLC-MS/MS approach for measuring the concentrations of oprozomib in plasma for the first time. This method was demonstrated to have good reproducibility, high accuracy, precision, and good linearity and had the advantages of short analysis time, simple sample preparation, and low cost. It showed the value in the pharmacokinetic study of oprozomib in rats.

## Figures and Tables

**Table 1:** The precision and accuracy of oprozomib in rat plasma (*n* = 5).

Analyte	Concentration (ng/mL)	Intraday		Interday	
		RSD (%)	RE (%)	RSD (%)	RE (%)
Oprozomib	1.0	4.7	−5.3	7.3	−1.7
	2.0	3.8	−14.9	6.6	−12.5
	40	3.1	1.7	4.9	6.5
	80	3.8	4.6	6.5	12.9

RSD: relative standard deviation; RE: relative error.

**Table 2:** Recovery and matrix effect of oprozomib in rat plasma (*n* = 5).

Analyte	Concentration added (ng/mL)	Recovery (%)		Matrix effect (%)	
		Mean ± SD	RSD (%)	Mean ± SD	RSD (%)
Oprozomib	2.0	85.1 ± 7.1	8.4	110.4 ± 11.5	10.4
	40	94.5 ± 3.5	3.7	109.7 ± 9.9	9.0
	80	96.1 ± 1.6	1.7	106.8 ± 8.8	8.3

SD: standard deviation; RSD: relative standard deviation.

**Table 3:** Stability results of oprozomib in plasma under different conditions (*n* = 5).

Analyte	Concentration added (ng/mL)	Room temperature, 3 h		Autosampler 10°C, 4 h		Three freeze-thaw		−80°C, 3 weeks	
		RSD (%)	RE (%)	RSD (%)	RE (%)	RSD (%)	RE (%)	RSD (%)	RE (%)
Oprozomib	2.0	6.2	10.7	6.7	−13.3	7.1	11.3	4.2	−0.2
	40	2.2	−1.2	3.7	−0.3	2.6	2.8	4.2	1.5
	80	6.3	8.5	3.6	1.1	6.3	13.7	7.6	13.5

RSD: relative standard deviation; RE: relative error.

**Table 4:** The main pharmacokinetic parameters of oprozomib in rat plasma after oral administration of oprozomib at a single dose of 21 mg/kg (*n* = 6, mean ± SD).

Parameters	Oprozomib
AUC_0⟶t_ (ng/mL•h)	134.6 ± 52.6
AUC_0⟶∞_ (ng/mL•h)	144.0 ± 55.5
*t*_1/2_ (h)	13.8 ± 8.3
*T*_max_ (h)	1.1 ± 0.3
CLz/F (L/h/kg)	172.9 ± 85.5
*C*_max_ (ng/mL)	46.5 ± 20.1

AUC: area under the plasma concentration-time curve; *t*_1/2_: elimination half time; *T*_max_: peak time; CLz/F: plasma clearance; *C*_max_: maximum plasma concentration. RSD: relative standard deviation; RE: relative error.

## Data Availability

The data used to support the study are available from the corresponding author upon reasonable request.
